# Genetic diversity and stability of the *porA* allele as a genetic marker in human *Campylobacter* infection

**DOI:** 10.1099/mic.0.031047-0

**Published:** 2009-12

**Authors:** A. J. Cody, M. J. C. Maiden, K. E. Dingle

**Affiliations:** 1 The Tinbergen Building, Department of Zoology, University of Oxford, South Parks Road, OX1 3PS, UK; 2 Nuffield Department of Clinical Laboratory Sciences, Oxford University, John Radcliffe Hospital, Oxford OX3 9DU, UK; 3 National Institute for Health Research, Oxford Biomedical Research Centre Programme, John Radcliffe Hospital, Oxford OX3 9DU, UK

## Abstract

The major outer-membrane protein (MOMP) of *Campylobacter jejuni* and *Campylobacter coli*, encoded by the *porA* gene, is extremely genetically diverse. Conformational MOMP epitopes are important in host immunity, and variation in surface-exposed regions probably occurs as a result of positive immune selection during infection. *porA* diversity has been exploited in genotyping studies using highly discriminatory nucleotide sequences to identify potentially epidemiologically linked cases of human campylobacteriosis. To understand the overall nature and extent of *porA* diversity and stability in *C. jejuni* and *C. coli* we investigated sequences in isolates (*n*=584) obtained from a defined human population (approx. 600 000) over a defined time period (1 year). A total of 196 distinct *porA* variants were identified. Regions encoding putative extracellular loops were the most variable in both nucleotide sequence and length. Phylogenetic analysis identified three *porA* allele clusters that originated in (i) predominantly *C. jejuni* and a few *C. coli*, (ii) solely *C. jejuni* or (iii) predominantly *C. coli* and a few *C. jejuni*. The stability of *porA* within an individual human host was investigated using isolates cultured longitudinally from 64 sporadic cases, 27 of which had prolonged infection lasting between 5 and 98 days (the remainder having illness of normal duration, 0–4 days), and 20 cases from family outbreaks. Evidence of mutation was detected in two patients with prolonged illness. Despite demonstrable positive immune selection in these two unusual cases, the persistence of numerous variants within the population indicated that the *porA* allele is a valuable tool for use in extended typing schemes.

## INTRODUCTION

Campylobacters are ubiquitous, being members of the gastrointestinal microbiota of poultry, wild birds, and farm and other animals. Whilst animal infection is usually asymptomatic, it provides sources of human infection via contaminated food products or untreated water, direct contact with animals, or contaminated environments (Hopkins *et al.*, 1984; Pebody *et al.*, 1997). Human disease, caused by invasion of the intestinal epithelial layer, can lead to localized inflammation and diarrhoea (Young *et al.*, 2007). The annual incidence of human campylobacteriosis in the UK is in excess of 340 000 cases (Kessel *et al.*, 2001), approximately 90 % of which are caused by *Campylobacter jejuni*, with the majority of the remainder attributed to *Campylobacter coli*. Although occasional large-scale outbreaks occur, the majority of cases are regarded as sporadic (Forbes *et al.*, 2009; Konkel *et al.*, 1999; Pebody *et al.*, 1997).

*C. jejuni* and *C. coli* are genetically diverse, even in genes encoding cellular metabolism, whose products are not surface exposed. Such ‘housekeeping’ loci have been used in multilocus sequence typing (MLST) schemes, data from which have been used to define bacterial population structures in a wide variety of bacteria including members of the genus *Campylobacter*. The *C. jejuni* and *C. coli* population structures comprise many clonal complexes (Dingle *et al.*, 2005, 2001), with at least 43 described to date (http://pubmlst.org/campylobacter). The diversity of *Campylobacter* cell-surface antigens has been exploited to develop a highly discriminatory 10-locus typing scheme, combining MLST data with sequences from *flaA, flaB* (encoding flagellin proteins) (Harrington *et al.*, 1997; Meinersmann *et al.*, 1997) and *porA* (encoding major outer-membrane protein, MOMP), which shows potential for the detection of epidemiologically linked cases (Dingle *et al.*, 2008).

The outer-membrane proteins of Gram-negative bacteria are porins, which are pore proteins that have a number of roles including regulation of cell membrane permeability to small molecules, adherence to host cells and antibiotic resistance (Buchanan, 1999; Cowan *et al.*, 1992; Jeanteur *et al.*, 1991; Kervella *et al.*, 1992; Moser *et al.*, 1997; Page *et al.*, 1989; Schroder & Moser, 1997). Therefore, they are sites of interaction of the bacterial cell with the environment and may play an important role in the adaptation of *Campylobacter* to various hosts. The *Campylobacter porA* gene consists of seven highly variable regions interspersed among conserved sequences. Deduced amino acid sequences comprise long irregular external loops connected by 18 *β*-strands and short periplasmic turns (Zhang *et al.*, 2000). These surface-exposed conformational epitopes appear to be responsible for MOMP antigenic variation, and evaluation of this antigen as a potential animal vaccine candidate has demonstrated its importance in conferring immunity (Huang *et al.*, 2007).

Our aims were (i) to characterize the extent of *porA* genetic diversity in *C. jejuni* and *C. coli* in a representative collection of human isolates of known population structure (defined by MLST), and (ii) to investigate *porA* stability during human infection for evidence of a role for immune selection in the generation of *porA* diversity, and in so doing further validate a previously published extended typing scheme (Dingle *et al.*, 2008).

## METHODS

### *Campylobacter* isolates.

*Campylobacter* isolates were obtained from the Clinical Microbiology Laboratory of the John Radcliffe Hospital, Oxford, which serves a population of approximately 600 000. A total of 620 human stool samples submitted between September 2003 and September 2004 were culture positive for *Campylobacter* species. Twenty-seven individuals provided two or more specimens, leaving *Campylobacter* isolates from 584 cases available for study. These were supplemented with isolates from a further 39 patients who submitted two or more stool samples between mid-September 2004 and July 2006, and isolates obtained from 10 family outbreaks. A family outbreak was defined as more than one individual from the same household, with the same surname, presenting with gastroenteritis within a 7 day period.

Isolates were stored at −70 °C in Tryptone Soy Broth plus glycerol (10 %) and cell-free DNA extracts were produced from a suspension of *Campylobacter* cells (cultured on charcoal cefoperazone deoxycholate agar plates) in molecular biology grade water (125 μl, Sigma Aldrich). The suspension was vortexed briefly to mix, heated at 100 °C for 10 min and debris was removed by centrifugation. The supernatant, containing chromosomal DNA, was removed and stored at −20 °C. The isolates were speciated using a previously published multiplex PCR assay (Wang *et al.*, 2002), with modifications as described by Dingle *et al.* (2002).

### Multilocus sequence typing.

Sequence typing of internal fragments of seven housekeeping genes (aspartase A, *aspA*; glutamine synthetase, *glnA*; citrate synthase, *gltA*; serine hydroxymethyltransferase, *glyA*; phosphoglucomutase, *pgm*; transketolase, *tkt* and ATP synthase *α* subunit, *uncA*) was performed as described previously, with minor modifications (Dingle *et al.*, 2005, 2001). Primers designed to amplify the relevant loci from both *C. jejuni* and *C. coli* isolates (Miller *et al.*, 2005) were substituted at loci that failed to produce amplicons in initial PCRs. Amplification products were purified by precipitation with 20 % polyethylene glycol/2.5 M NaCl (Embley, 1991). Nucleotide sequencing was performed at least once on each DNA strand using the primers employed for the initial amplification and BigDye Ready Reaction Mix (Version 3, Applied Biosystems) at a concentration 32-fold lower than that described in the manufacturer's instructions. Existing and new alleles, sequence types (ST) and clonal complexes were assigned using the MLST database located at http://pubmlst.org/campylobacter/.

### Antigen sequence determination.

Nucleotide sequences were obtained from three antigen genes, the short variable region (SVR) of (i) *flaA* and (ii) *flaB*, encoding flagellin protein (Harrington *et al.*, 1997; Meinersmann *et al.*, 1997), and (iii) *porA*, encoding the MOMP (Dingle *et al.*, 2008). A 321 bp sequence containing the *flaA* SVR was obtained for each isolate using oligonucleotide primers FLA4F, FLA242FU and FLA625RU (Meinersmann *et al.*, 1997). The corresponding *flaB* SVR sequences were amplified and sequenced using oligonucleotide primers Bup and A6 (Harrington *et al.*, 1997). In both instances, the amplification and sequencing reaction conditions were as for MLST. SVR allele numbers and peptide numbers for both genes were assigned using the *flaA* and *flaB* database, accessible at http://pubmlst.org/campylobacter/flaA and newly identified sequences were deposited there.

Sequences from *porA* were obtained using primers MOMP3 and MOMP2 (Dingle *et al.*, 2008). Amplification reactions (25 μl) were prepared of equivalent composition to those described above. Reaction conditions and the approach used for trimming sequences to the correct length for database queries are described at http://pubmlst.org/campylobacter/momp. Allele numbers were also assigned by querying this database.

### Data analysis.

Codon-aligned *porA* nucleotide and amino acid sequences were constructed using the program Seqlab in gcg (Wisconsin Package version 10.3, Accelerys) to identify conserved and variable regions of the allele. Sites in the sequence alignment at which positive immune selection had occurred were detected by use of snap.pl (Korber, 2000; Ota & Nei, 1994), available at http://www.hiv.lanl.gov. This program calculates synonymous (*d*_S_) and non-synonymous (*d*_N_) substitution rates by the method of Nei & Gojobori (1986) and incorporates a statistic developed by Ota & Nei (1994). Sites of positive immune selection were defined as those at which a greater number of non-synonymous to synonymous substitutions were identified.

The association between clonal complex and *porA* allele was measured using a permutation test in which actual data and 1000 random associations were compared.

Phylogenetic relationships among *porA* alleles were determined using ClonalFrame (Didelot & Falush, 2007). Sequence data from alleles were aligned using the program muscle, available at http://www.ebi.ac.uk/Tools/muscle/index.html, to obtain an input file for ClonalFrame. A 75 % consensus tree was constructed from five convergent replicate trees, by setting both the number of burn-in iterations and the number of Monte Carlo Markov chain (MCMC) iterations to 50 000. Convergence of the MCMC between replicates was determined by a Gelman & Rubin (1992) statistic below 1.2 for each parameter.

## RESULTS

### Genetic diversity in a representative collection of isolates

The isolate collection comprised 540 *C. jejuni* (92.62 %), 43 *C. coli* (7.38 %) and a single *Campylobacter upsaliensis* isolate*,* which was excluded from further analysis. A total of 167 STs were represented at least once among these isolates; 119 were members of 27 clonal complexes (71.3 %) with the remaining 48 ST unassigned (28.7 %) (Supplementary Table S1). The 6 most common clonal complexes contained 55.9 % of the isolates.

Allele sequences for three antigen genes, the *flaA* and *flaB* SVR and *porA*, were determined for 575, 568, and 569 isolates respectively. The ratio of the number of unique nucleotide sequences to the number of unique peptide sequences for the *flaA* SVR was 130 : 43 (3.023) and *flaB* SVR was 111 : 38 (2.921). Sequences for *porA* represented 196 alleles, encoding 180 different peptides (ratio 1.088) varying in length from 582 to 702 nucleotides. The frequency of the different *porA* alleles within the dataset was highly variable (Supplementary Fig. S1), with allele 1 occurring in 62 isolates (10.9 %) whilst the majority of alleles (*n*=135) occurred only once.

**Fig. 1. f1:**
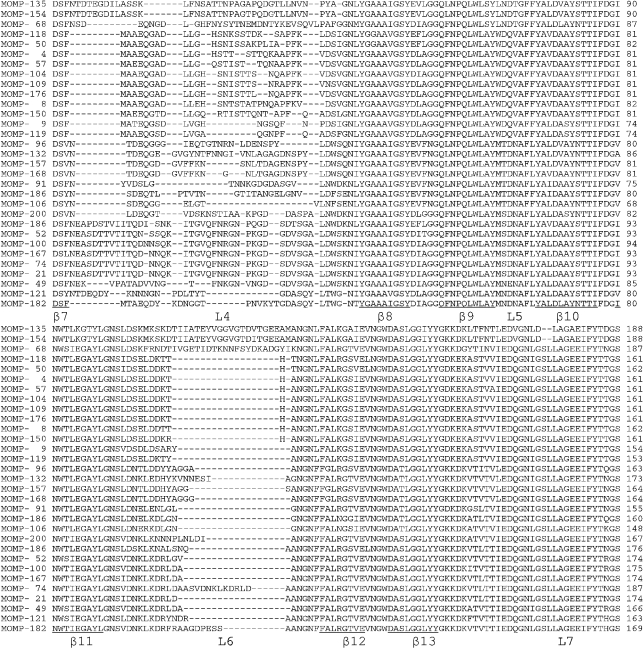

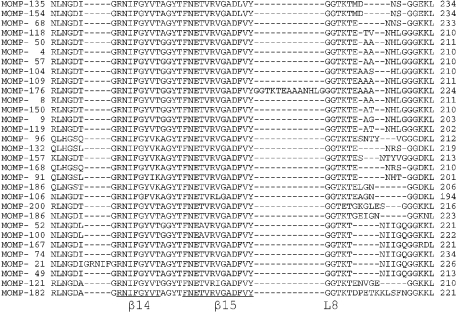
Alignment of 31 deduced MOMP sequences chosen to represent the diversity observed among 180 peptides identified in human *Campylobacter* isolates. Protein variant number is indicated to the left and amino acid length to the right of each sequence. Gaps are indicated by -. Bold lines below sequence blocks indicate regions predicted to form *β*-barrels, and regions encoding putative external loops are labelled L4 to L8, as described by Zhang *et al.* (2000).

**Fig. 2. f2:**
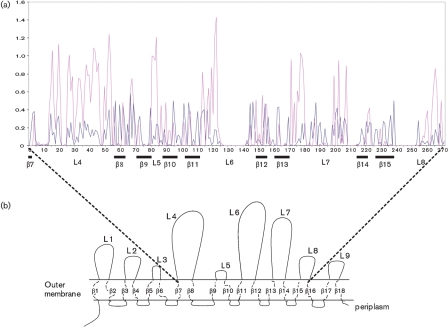
Distribution of synonymous and non-synonymous mutations across the *Campylobacter porA* allele in relation to the protein structure predicted by Zhang *et al.* (2000). (a) Plot demonstrating the average for each codon for all pairwise comparisons for indels, synonymous and non-synonymous mutations calculated from 196 *porA* nucleotide alleles by SNAP.pl (Korber, 2000; Ota & Nei, 1994). Synonymous mutations are indicated by the blue line and non-synonymous mutations by the pink line. *x*-axis annotation indicates codon number and related predicted protein structure. (b) Schematic representation of the *Campylobacter* MOMP indicating putative intra-membrane *β*-barrels and surface-exposed loops.

Nucleotide and amino acid sequences of the *porA* variants were codon-aligned using Seqlab in gcg. Allele *porA*-149 (MOMP-135) was used as a point of reference with which initial comparisons were made, as it was one of the longest. The alignment demonstrated that diversity had been generated by point mutations, duplications and insertions or deletions of novel sequence segments (Fig. 1). Comparison of this alignment with the secondary structure predicted for the MOMP by Zhang *et al.* (2000) identified regions encoding five putative surface-exposed loops (Fig. 2) encoded by the internal fragment of the *porA* gene included in this study. The sequence started at the putative distal aspect of the seventh *β*-barrel, immediately prior to external loop 4, and ended at the final amino acid of external loop 8.

Sites in the sequence alignment at which positive immune selection had occurred (*d*_N_ substitution rate>*d*_S_ rate) were identified (Fig. 2). An excess of non-synonymous substitutions was present within external loops 4, 5 and 6, and parts of loop 7 and loop 8, and absent in most of the transmembrane domains. Parts of the sequences of loops 6, 7 and 8 where a long insertion sequence was identified in three or fewer of the alleles demonstrated neither synonymous nor non-synonymous mutations.

### Putative structural variation

Sequence variation in the transmembrane domains and external loops was assessed among the 180 different MOMP variants. The transmembrane domains were conserved in length and varied relatively little in peptide sequence (Fig. 1). Non-synonymous mutations did occur, but these were predominantly conservative or semi-conservative amino acid substitutions. In contrast, most of the extracellular loops varied markedly (Fig. 1).

Most variation in both putative amino acid sequence and length was present in loop 4. Extracellular loop 5 showed little sequence diversity and no variation in length. The longest sequence insertions (60 bp) were detected in putative extracellular loop 6 of two *C. coli* isolates (MOMP-135 and MOMP-134) (Fig. 1). A novel insertion sequence of 20 aa was found at the same position in a single *C. jejuni* isolate, belonging to the ST-21 complex (MOMP-68). These three alleles were rare, each occurring only once in the isolate collection. MOMP-74, which occurred twice, had a 13 aa duplication in this region. Within loop 7, two variants had a 2 aa deletion (MOMP-135 and MOMP-154), and one variant a 5 aa duplication (MOMP-21). These three alleles also occurred only once. Loop 8 varied in both sequence and length, the latter predominantly as a result of a 13 aa repeat in MOMP-176, which occurred only once in the dataset.

### Phylogenetic relationships among *porA* alleles

**Fig. 3. f3:**
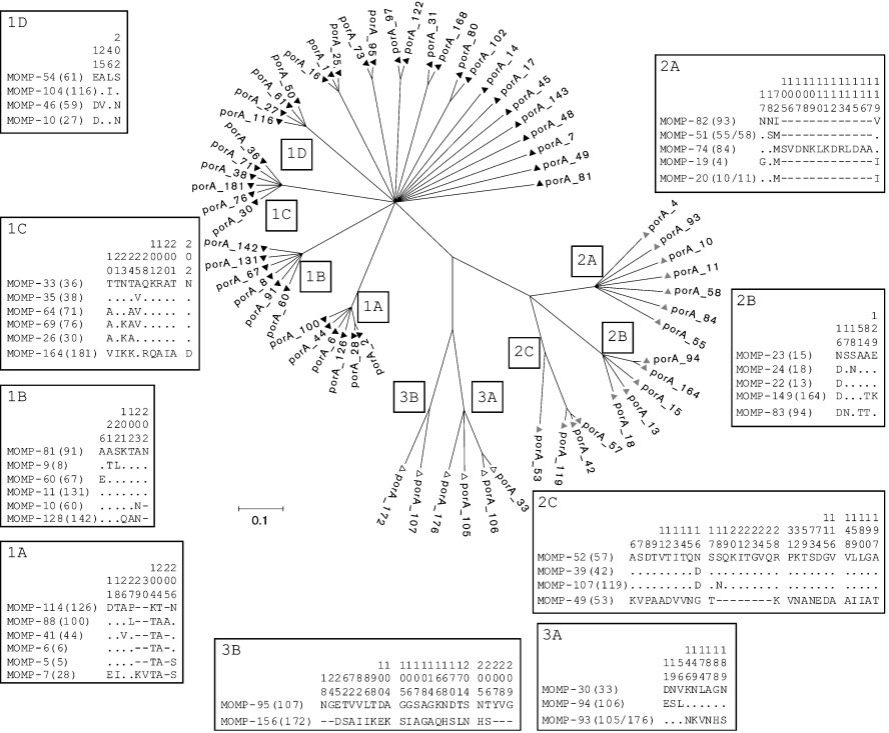
ClonalFrame tree constructed using the 63 *porA* alleles occurring more than once in the dataset. Amino acid sequence alignments indicating polymorphic sites appear alongside each clade. Numbers in parentheses indicate the nucleotide sequence encoding the protein sequence indicated. Numbers above the alignment indicate the position within each clade alignment at which polymorphisms occur.

Three major sequence groups were detected by phylogenetic analysis of the *porA* allele sequences, constructed using a subset of the most commonly identified 63 *porA* alleles (each occurring at least twice) to simplify interpretation (Fig. 3). When the full dataset of 196 *porA* alleles was analysed, sequence group 1 contained 118 alleles (60.02 %) subdivided into 85 clades, 74 of which did not share a recent common ancestor with other members of the group (Supplementary Fig. S2). Sequence group 2 contained 44 (22.45 %) alleles subdivided into 15 clades, 8 of which had no recent common ancestors with other members of the group. Group 3 had 34 (17.35 %) alleles which formed 3 clades. A further 7 (3.57 %) sequences had no common ancestor with any of the sequence groups. Groups 1 and 3 contained alleles from both *C. jejuni* and *C. coli* isolates (Supplementary Fig. S2). Group 1 was mostly *C. jejuni*, with 2 *C. coli* alleles, and conversely group 3 was mostly *C. coli*, with alleles from 3 *C. jejuni* isolates. Group 2 was composed solely of sequences from *C. jejuni*. All three groups were well represented among the data since isolates from each group occurred with a frequency of 10 or greater (Supplementary Fig. S1). There was only one example of *C. coli* and *C. jejuni* isolates sharing the same *porA* allele: *porA*-33 occurred 10 times in *C. coli* ST-828, but once in *C. jejuni* ST-257 and ST-53. The ST-53 isolate was also antigenically identical to 9 of the 10 *C. coli* ST-828 isolates at the *flaA* and *flaB* SVRs. Variations in the amino acid sequences of the extracellular loops were mapped onto the tree (Fig. 3).

### Association between clonal complex and MOMP

Among the 569 isolates there were 180 MOMP variants and 27 clonal complexes, which occurred in a total of 228 combinations, 154 of which occurred only once. When all the data were considered together, MOMP had a non-random association with clonal complex (*P*<0.01). For example, nine of the MOMP variants (each occurring 10 times or more) were associated predominantly with 11 clonal complexes (Supplementary Fig. S3). However, these relationships were not exclusive and a single MOMP variant was not predictive of clonal complex. For example, MOMP-1 was associated mainly with ST-257 complex, ST-574 complex and ST-658 complex. Conversely, a given clonal complex was not predictive of MOMP variant, individual clonal complexes being associated with more than one variant; for example ST-21 complex was most commonly associated with MOMP-4, 6, 10 and 13.

### Stability of *porA* during prolonged infection

Evidence of *porA* mutation during prolonged infection was found in two patients with prolonged illness (5–34 days), each of whom had submitted three *C. jejuni* isolates. In patient 55, the third isolate, collected 16 days after the first, had a G to A transition at the second nucleic acid of codon 170 of the allele. This resulted in a glycine to aspartic acid substitution, a non-conservative change located in putative surface-exposed loop 7 of the protein. The MLST type of this isolate was ST-45, the central genotype of ST-45 complex, which is commonly identified in human infections (Dingle *et al.*, 2002).

In patient 59, the third isolate, collected 27 days after the first, had a histidine insertion created by a CAT repeat. The insertion was located after codon 204 of the allele in putative surface-exposed loop 8. This isolate was ST-843, a member of ST-21 complex, the most frequent among human infections (Dingle *et al.*, 2002). The results were confirmed for both patients 55 and 59 from a minimum of 9 individual colonies. In all cases, DNA templates were prepared from a sweep of bacterial growth rather than an individual colony, to ensure amplification of the predominant genotype in any mixed cultures. No synonymous nucleotide changes were observed in isolates from either patient.

In a further three patients (60, 63 and 64), with illness of longer than normal duration, the second isolate cultured was genetically distinct from the first at all 10 loci (Supplementary Table S2). This suggests either a co-infection or, due to the long time intervals between the isolates (31, 56 and 98 days), subsequent independent infections. However, there was no evidence of *porA* mutation in 59 samples from 24 other patients during the course of their prolonged infections, which ranged in length from 6 to 34 days (mean sampling interval 12.6 days). Neither was any difference in the 10-locus profile identified in any of the 46 isolates obtained from 22 patients that provided more than one sample per day, at some point during the course of their illness. These control samples validated the methodology and reproducibility of results.

The MLST and antigenic profiles of 78 isolates from 37 patients with typical duration of illness (0–4 days; mean sampling interval 1.1 days) remained unchanged, although one patient was simultaneously infected with two unrelated *Campylobacter* strains. Twenty isolates obtained from 10 family outbreaks did not vary among individuals of the same household, but in one outbreak the individuals were infected by genotypically different isolates (Supplementary Table S3).

## DISCUSSION

A highly discriminatory typing scheme has been developed to detect potentially linked cases of human campylobacteriosis. This typing scheme extends the MLST approach by exploiting the extensive genetic diversity of *Campylobacter porA*, and the SVRs of the flagellin genes *flaA* and *flaB* (Dingle *et al.*, 2008). However, the potential for positive immune selection at putative surface-exposed regions of the PorA MOMP raises questions regarding the stability of the *porA* locus, and hence its suitability for inclusion in such schemes. This study has assessed the nature and extent of *porA* genetic diversity in *C. jejuni* and *C. coli* and the stability of the locus during human infection. It provides important validation for the extended typing scheme.

The level of diversity we observed at the *porA* locus was far greater than that at the housekeeping loci used in MLST, and at the SVRs of *flaA* and *flaB*, which encode flagellin, a major immunodominant antigen in humans (Nachamkin & Yang, 1989, 1992). The ratio of *porA* alleles to peptides was approximately threefold lower than that observed for the *flaA* and *flaB* SVRs. Among the *porA* alleles, almost every distinct nucleotide sequence encoded a novel peptide, with non-synonymous substitutions in excess of synonymous substitutions. This provides evidence of strong positive immune selection, especially in putative surface-exposed loops. The observation from this collection of human isolates is supported by the *porA* database (http://pubmlst.org/campylobacter/momp), which contains 829 *porA* allele sequences, encoding 770 protein variants, a ratio of 1.07 (as of 8 May, 2009). Also, when published *porA* sequences from a total of 106 *C. jejuni* and *C. coli* isolates available from GenBank (Clark *et al.*, 2007; Huang *et al.*, 2005; Zhang *et al.*, 2000) were compared with those from the present isolate collection, 25 alleles were common to both datasets. Of these, 17 (68 %) were observed more than once in our dataset (Supplementary Fig. S1), showing that these variants are, at least to some degree, persistent in the population.

The *porA* allele sequences formed three major phylogenetic clusters (Fig. 3), two containing sequences derived from both *C. jejuni* and *C. coli* isolates (groups 1 and 3) and the third containing only sequences derived from *C. jejuni* isolates (group 2). A previous study also identified three *porA* groups, but in this case, only that corresponding to group 3 contained both *C. coli* and *C. jejuni* sequences (Clark *et al.*, 2007). Recombination between the two species was suggested as an explanation for this observation, and independent evidence of recombination among *C. jejuni* and *C. coli* isolates has been described (Sheppard *et al.*, 2008). Estimates have indicated that recombination between *C. jejuni* and *C. coli* generates diversity at twice the rate of *de novo* mutation (Wilson *et al.*, 2009). Our results support these findings, as a small number of *C. coli* isolates were present in a mostly *C. jejuni* group and vice versa (Fig. 3). However, in contrast to the *flaA* and *flaB* SVRs, (Dingle *et al.*, 2002) only a single *porA* allele (*porA-*33*)* was identified in isolates of both *C. jejuni* and *C. coli*. Therefore, whilst uncommon in this sample of isolates, there is evidence of recombination between the two species involving *porA*.

Population analysis of *C. jejuni* isolates from diverse hosts has demonstrated associations between certain clonal complexes and isolation sources (Dingle *et al.*, 2002), suggesting that some genotypes are particularly well adapted to certain host environments. MLST was subsequently used to predict the reservoirs in which particular strains originated (McCarthy *et al.*, 2007; Sheppard *et al.*, 2009). A number of clonal complexes are relatively homogeneous in their heat-stable serotype and FlaA SVR variant (Dingle *et al.*, 2002). In addition, the findings presented here demonstrated a non-random association between clonal complexes and *porA* variants (*P*<0.01). The host immune response has been suggested to play a role in defining the more antigenically homogeneous clonal complexes, and this could also reflect niche adaptation. For example, ST-45 and ST-257 complexes are human and chicken associated (Dingle *et al.*, 2002), and predominantly MOMP-1 and MOMP-41 (alleles for both these proteins were found in sequence group 1: MOMP-41 in clade 1A and MOMP-1 in a clade of its own; Fig. 3). Alignment of these sequences demonstrates that they differ predominantly at loop 4. A definitive study on MOMP host association would require MLST and *porA* data for isolates from a wide variety of hosts, and we cannot extrapolate from a study focusing on human isolates alone; however, published data from Clark *et al.* (2007), Huang *et al.* (2005) and Zhang *et al.* (2000) indicated that MOMP-1 and MOMP-41 were human and chicken associated. A complicating factor in exploring these relationships for all *C. jejuni* and *C. coli* isolates may be their ability to colonize multiple hosts and thereby undergo exposure to many different immune responses.

*Campylobacter*-associated diarrhoea in humans generally lasts 3 to 4 days, but shedding during convalescence can continue for several weeks, carriage being reported in 16 % of individuals, for a median time of 31 days (Kapperud *et al.*, 1992). The *porA* allele has been used to enhance isolate discrimination and identify potentially epidemiologically linked cases (Dingle *et al.*, 2008). It was therefore useful to confirm that *porA* was sufficiently stable for this purpose, and not overdiscriminatory due to frequent ‘within-patient’ mutations. In the present study, the *porA* allele was stable in the majority of patients examined and in 10 family outbreaks. Isolates collected longitudinally from two patients with prolonged infection underwent mutation within putative surface-exposed loops. The patients were still suffering diarrhoea when these mutations occurred, but clinical data on their immune status were unavailable. Convalescent stool samples for patients with illness of normal duration were not available, but these would be unlikely to yield a high frequency of isolates with new *porA* mutations, since we anticipate that these would be associated with immune escape and continued symptomatic illness. Conformational epitopes are important in immunity to *Campylobacter* (Cawthraw *et al.*, 2002; Huang *et al.*, 2007); therefore, the within-patient mutations detected here may result from continued *Campylobacter* exposure to the patients' immune response.

Campylobacters are widely distributed in poultry, wild birds, and farm and other animals (Corry & Atabay, 2001; Jones, 2001; Stanley & Jones, 2003). Poultry are considered the major reservoir for human infection (Friedman *et al.*, 2004; Kapperud *et al.*, 2003; Wingstrand *et al.*, 2006) and once colonization of a chicken has occurred, *Campylobacter* can remain in a bird's caecum for its entire life (Wagenaar *et al.*, 2006). A number of surface-exposed *Campylobacter* proteins have been identified as immunogenic in humans, including MOMP (Cawthraw *et al.*, 2002), and among humans, circulating antibodies can be detected 6 to 7 days after the onset of symptoms (Newell & Nachamkin, 1992). The within-patient mutations detected here may therefore indicate immune evasion, since they occurred in the patient after we expect antibody production to have occurred. Preliminary data from chickens indicated that *porA* alleles are stable during long-term colonization (F. M. Colles, personal communication); therefore a similar process of immune selection may be absent in chickens. Further studies are required for confirmation of different immune pressures in human and non-human hosts.

In conclusion, the variability of the *porA* surface loops provides evidence that immune selection strongly influences the diversity of this locus. However, our data validate the use of the *porA* locus in extended typing schemes, since with the exception of two unusual cases, this highly diverse locus was stable during individual infections and family outbreaks.

## Abbreviations

- MLST, multilocus sequence typing
- MOMP, major outer-membrane protein
- ST, sequence type
- SVR, short variable region

## References

[r1] Buchanan, S. K. (1999). Beta-barrel proteins from bacterial outer membranes: structure, function and refolding. Curr Opin Struct Biol 9, 455–461. 10449368 10.1016/S0959-440X(99)80064-5

[r2] Cawthraw, S. A., Feldman, R. A., Sayers, A. R. & Newell, D. G. (2002). Long-term antibody responses following human infection with *Campylobacter jejuni*. Clin Exp Immunol 130, 101–106. 12296859 10.1046/j.1365-2249.2002.01966.x PMC1906500

[r3] Clark, C. G., Beeston, A., Bryden, L., Wang, G., Barton, C., Cuff, W., Gilmour, M. W. & Ng, L. K. (2007). Phylogenetic relationships of *Campylobacter jejuni* based on *porA* sequences. Can J Microbiol 53, 27–38. 17496947 10.1139/w06-099

[r4] Corry, J. E. & Atabay, H. I. (2001). Poultry as a source of *Campylobacter* and related organisms. Symp Ser Soc Appl Microbiol 96S–114S. 10.1046/j.1365-2672.2001.01358.x 11422565

[r5] Cowan, S. W., Schirmer, T., Rummel, G., Steiert, M., Ghosh, R., Pauptit, R. A., Jansonius, J. N. & Rosenbusch, J. P. (1992). Crystal structures explain functional properties of two *E. coli* porins. Nature 358, 727–733. 1380671 10.1038/358727a0

[r6] Didelot, X. & Falush, D. (2007). Inference of bacterial microevolution using multilocus sequence data. Genetics 175, 1251–1266. 17151252 10.1534/genetics.106.063305 PMC1840087

[r7] Dingle, K. E., Colles, F. M., Wareing, D. R. A., Ure, R., Fox, A. J., Bolton, F. J., Bootsma, H. J., Willems, R. J. L., Urwin, R. & Maiden, M. C. J. (2001). Multilocus sequence typing system for *Campylobacter jejuni*. J Clin Microbiol 39, 14–23. 11136741 10.1128/JCM.39.1.14-23.2001 PMC87672

[r8] Dingle, K. E., Colles, F. M., Ure, R., Wagenaar, J., Duim, B., Bolton, F. J., Fox, A. J., Wareing, D. R. A. & Maiden, M. C. J. (2002). Molecular characterisation of *Campylobacter jejuni* clones: a rational basis for epidemiological investigations. Emerg Infect Dis 8, 949–955. 12194772 10.3201/eid0809.02-0122 PMC2732546

[r9] Dingle, K. E., Colles, F. M., Falush, D. & Maiden, M. C. (2005). Sequence typing and comparison of population biology of *Campylobacter coli* and *Campylobacter jejuni*. J Clin Microbiol 43, 340–347. 15634992 10.1128/JCM.43.1.340-347.2005 PMC540151

[r10] Dingle, K. E., McCarthy, N. D., Cody, A. J., Peto, T. E. & Maiden, M. C. (2008). Extended sequence typing of *Campylobacter* spp., United Kingdom. Emerg Infect Dis 14, 1620–1622. 18826829 10.3201/eid1410.071109 PMC2609887

[r11] Embley, T. M. (1991). The linear PCR reaction: a simple and robust method for sequencing amplified rRNA genes. Lett Appl Microbiol 13, 171–174. 1370053 10.1111/j.1472-765x.1991.tb00600.x

[r12] Forbes, K. J., Gormley, F. J., Dallas, J. F., Labovitiadi, O., MacRae, M., Owen, R. J., Richardson, J., Strachan, N. J., Cowden, J. M. & other authors (2009). *Campylobacter* immunity and coinfection following a large outbreak in a farming community. J Clin Microbiol 47, 111–116. 19005146 10.1128/JCM.01731-08 PMC2620832

[r13] Friedman, C. R., Hoekstra, R. M., Samuel, M., Marcus, R., Bender, J., Shiferaw, B., Reddy, S., Ahuja, S. D., Helfrick, D. L. & other authors (2004). Risk factors for sporadic *Campylobacter* infection in the United States: a case-control study in FoodNet sites. Clin Infect Dis 38 (Suppl. 3), S285–S296. 15095201 10.1086/381598

[r14] Gelman, A. & Rubin, D. B. (1992). Inference from iterative simulation using mulitple sequences. Stat Sci 7, 457–472.

[r15] Harrington, C. S., Thomson Carter, F. M. & Carter, P. E. (1997). Evidence for recombination in the flagellin locus of *Campylobacter jejuni*: implications for the flagellin gene typing scheme. J Clin Microbiol 35, 2386–2392. 9276421 10.1128/jcm.35.9.2386-2392.1997 PMC229973

[r16] Hopkins, R. S., Olmsted, R. & Istre, G. R. (1984). Endemic *Campylobacter jejuni* infection in Colorado: identified risk factors. Am J Public Health 74, 249–250. 6696155 10.2105/ajph.74.3.249 PMC1651452

[r17] Huang, S., Luangtongkum, T., Morishita, T. Y. & Zhang, Q. (2005). Molecular typing of *Campylobacter* strains using the *cmp* gene encoding the major outer membrane protein. Foodborne Pathog Dis 2, 12–23. 15992295 10.1089/fpd.2005.2.12

[r18] Huang, S., Sahin, O. & Zhang, Q. (2007). Infection-induced antibodies against the major outer membrane protein of *Campylobacter jejuni* mainly recognize conformational epitopes. FEMS Microbiol Lett 272, 137–143. 17521366 10.1111/j.1574-6968.2007.00752.x

[r19] Jeanteur, D., Lakey, J. H. & Pattus, F. (1991). The bacterial porin superfamily: sequence alignment and structure prediction. Mol Microbiol 5, 2153–2164. 1662760 10.1111/j.1365-2958.1991.tb02145.x

[r20] Jones, K. (2001). Campylobacters in water, sewage and the environment. Symp Ser Soc Appl Microbiol 68S–79S. 10.1046/j.1365-2672.2001.01355.x 11422562

[r21] Kapperud, G., Lassen, J., Ostroff, S. M. & Aasen, S. (1992). Clinical features of sporadic *Campylobacter* infections in Norway. Scand J Infect Dis 24, 741–749. 1287808 10.3109/00365549209062459

[r22] Kapperud, G., Espeland, G., Wahl, E., Walde, A., Herikstad, H., Gustavsen, S., Tveit, I., Natas, O., Bevanger, L. & Digranes, A. (2003). Factors associated with increased and decreased risk of *Campylobacter* infection: a prospective case-control study in Norway. Am J Epidemiol 158, 234–242. 12882945 10.1093/aje/kwg139

[r23] Kervella, M., Fauchere, J. L., Fourel, D. & Pages, J. M. (1992). Immunological cross-reactivity between outer membrane pore proteins of *Campylobacter jejuni* and *Escherichia coli*. FEMS Microbiol Lett 78, 281–285. 1337053 10.1016/0378-1097(92)90041-l

[r24] Kessel, A. S., Gillespie, I. A., O'Brien, S. J., Adak, G. K., Humphrey, T. J. & Ward, L. R. (2001). General outbreaks of infectious intestinal disease linked with poultry, England and Wales, 1992–1999. Commun Dis Public Health 4, 171–177. 11732355

[r25] Konkel, M. E., Gray, S. A., Kim, B. J., Garvis, S. G. & Yoon, J. (1999). Identification of the enteropathogens *Campylobacter jejuni* and *Campylobacter coli* based on the *cadF* virulence gene and its product. J Clin Microbiol 37, 510–517. 9986804 10.1128/jcm.37.3.510-517.1999 PMC84446

[r26] Korber, B. (2000). HIV signature and sequence variation analysis. In *Computational Analysis of HIV Molecular Sequences*, pp. 55–72. Edited by A. G. Rodrigo & G. H. Learn. Dordrecht, Netherlands: Kluwer.

[r27] McCarthy, N. D., Colles, F. M., Dingle, K. E., Bagnall, M. C., Manning, G., Maiden, M. C. & Falush, D. (2007). Host-associated genetic import in *Campylobacter jejuni*. Emerg Infect Dis 13, 267–272. 17479890 10.3201/eid1302.060620 PMC2063414

[r28] Meinersmann, R. J., Helsel, L. O., Fields, P. I. & Hiett, K. L. (1997). Discrimination of *Campylobacter jejuni* isolates by *fla* gene sequencing. J Clin Microbiol 35, 2810–2814. 9350739 10.1128/jcm.35.11.2810-2814.1997 PMC230067

[r29] Miller, W. G., On, S. L., Wang, G., Fontanoz, S., Lastovica, A. J. & Mandrell, R. E. (2005). Extended multilocus sequence typing system for *Campylobacter coli*, *C. lari*, *C. upsaliensis*, and *C. helveticus*. J Clin Microbiol 43, 2315–2329. 15872261 10.1128/JCM.43.5.2315-2329.2005 PMC1153752

[r30] Moser, I., Schroeder, W. & Salnikow, J. (1997). *Campylobacter jejuni* major outer membrane protein and a 59-kDa protein are involved in binding to fibronectin and INT 407 cell membranes. FEMS Microbiol Lett 157, 233–238. 9435102 10.1111/j.1574-6968.1997.tb12778.x

[r31] Nachamkin, I. & Yang, X. H. (1989). Human antibody response to *Campylobacter jejuni* flagellin protein and a synthetic N-terminal flagellin peptide. J Clin Microbiol 27, 2195–2198. 2584372 10.1128/jcm.27.10.2195-2198.1989 PMC266992

[r32] Nachamkin, I. & Yang, X. H. (1992). Local immune responses to the *Campylobacter* flagellin in acute *Campylobacter* gastrointestinal infection. J Clin Microbiol 30, 509–511. 1371521 10.1128/jcm.30.2.509-511.1992 PMC265089

[r33] Nei, M. & Gojobori, T. (1986). Simple methods for estimating the numbers of synonymous and nonsynonymous nucleotide substitutions. Mol Biol Evol 3, 418–426. 3444411 10.1093/oxfordjournals.molbev.a040410

[r34] Newell, D. G. & Nachamkin, I. (1992). Immune responses directed against *Campylobacter jejuni*. In *Campylobacter jejuni: Current Status and Future Trends*, pp. 201–206. Edited by I. Nachamkin, M. J. Blaser & L. S. Tompkins. Washington, DC: American Society for Microbiology.

[r35] Ota, T. & Nei, M. (1994). Variance and covariances of the numbers of synonymous and nonsynonymous substitutions per site. Mol Biol Evol 11, 613–619. 8078400 10.1093/oxfordjournals.molbev.a040140

[r36] Page, W. J., Huyer, G., Huyer, M. & Worobec, E. A. (1989). Characterization of the porins of *Campylobacter jejuni* and *Campylobacter coli* and implications for antibiotic susceptibility. Antimicrob Agents Chemother 33, 297–303. 2543277 10.1128/aac.33.3.297 PMC171482

[r37] Pebody, R. G., Ryan, M. J. & Wall, P. G. (1997). Outbreaks of *Campylobacter* infection: rare events for a common pathogen. Commun Dis Rep CDR Rev 7, R33–R37. 9080726

[r38] Schroder, W. & Moser, I. (1997). Primary structure analysis and adhesion studies on the major outer membrane protein of *Campylobacter jejuni*. FEMS Microbiol Lett 150, 141–147. 9163918 10.1111/j.1574-6968.1997.tb10362.x

[r39] Sheppard, S. K., McCarthy, N. D., Falush, D. & Maiden, M. C. (2008). Convergence of *Campylobacter* species: implications for bacterial evolution. Science 320, 237–239. 18403712 10.1126/science.1155532

[r40] Sheppard, S. K., Dallas, J. F., Strachan, N. J., MacRae, M., McCarthy, N. D., Wilson, D. J., Gormley, F. J., Falush, D., Ogden, I. D. & other authors (2009). *Campylobacter* genotyping to determine the source of human infection. Clin Infect Dis 48, 1072–1078. 19275496 10.1086/597402 PMC3988352

[r41] Stanley, K. & Jones, K. (2003). Cattle and sheep farms as reservoirs of *Campylobacter*. J Appl Microbiol 94 (Suppl), 104S–113S. 12675942 10.1046/j.1365-2672.94.s1.12.x

[r42] Wagenaar, J. A., Mevius, D. J. & Havelaar, A. H. (2006). *Campylobacter* in primary animal production and control strategies to reduce the burden of human campylobacteriosis. Rev Sci Tech 25, 581–594. 17094699

[r43] Wang, G., Clark, C. G., Taylor, T. M., Pucknell, C., Barton, C., Price, L., Woodward, D. L. & Rodgers, F. G. (2002). Colony multiplex PCR assay for identification and differentiation of *Campylobacter jejuni, C. coli, C. lari, C. upsaliensis*, and *C. fetus subsp. fetus*. J Clin Microbiol 40, 4744–4747. 12454184 10.1128/JCM.40.12.4744-4747.2002 PMC154608

[r44] Wilson, D. J., Gabriel, E., Leatherbarrow, A. J., Cheesbrough, J., Gee, S., Bolton, E., Fox, A., Hart, C. A., Diggle, P. J. & Fearnhead, P. (2009). Rapid evolution and the importance of recombination to the gastroenteric pathogen *Campylobacter jejuni*. Mol Biol Evol 26, 385–397. 19008526 10.1093/molbev/msn264 PMC2639114

[r45] Wingstrand, A., Neimann, J., Engberg, J., Nielsen, E. M., Gerner-Smidt, P., Wegener, H. C. & Molbak, K. (2006). Fresh chicken as main risk factor for campylobacteriosis, Denmark. Emerg Infect Dis 12, 280–285. 16494755 10.3201/eid1202.050936 PMC3373097

[r46] Young, K. T., Davis, L. M. & Dirita, V. J. (2007). *Campylobacter jejuni*: molecular biology and pathogenesis. Nat Rev Microbiol 5, 665–679. 17703225 10.1038/nrmicro1718

[r47] Zhang, Q., Meitzler, J. C., Huang, S. & Morishita, T. (2000). Sequence polymorphism, predicted secondary structures, and surface-exposed conformational epitopes of *Campylobacter* major outer membrane protein. Infect Immun 68, 5679–5689. 10992471 10.1128/iai.68.10.5679-5689.2000 PMC101523

